# Extracellular Alpha-Satellite DNA in Human Plasma as a Candidate Biomarker for Bladder Cancer Detection: Preliminary Evidence Using Digital PCR

**DOI:** 10.3390/ijms27156834

**Published:** 2026-07-30

**Authors:** Nunzia Santini, Alfredo Procino, Sven Ljubić, Damir Đermić, Đurđica Ugarković, Isidoro Feliciello

**Affiliations:** 1 Department of Clinical Medicine and Surgery, University of Naples Federico II, 80131 Naples, Italy; 2 Department of Molecular Biology, Ruder Boškovic Institute,10000 Zagreb, Croatia; sven.ljubic@irb.hr (S.L.); dermic@irb.hr (D.Đ.);

**Keywords:** bladder cancer, liquid biopsy, circulating cell-free DNA, extracellular DNA, alpha-satellite DNA, hASAT, digital PCR, ROC analysis, biomarker, non-muscle-invasive bladder cancer

## Abstract

Bladder cancer (BC) is a common urological malignancy that lacks the non-invasive biomarkers that would make it suitable for early diagnosis. Human alpha-satellite DNA (hASAT) is a tandemly repeated centromeric/pericentromeric DNA family associated with chromosomal stability and cancer-related genomic instability. We quantified extracellular hASAT (ec-hASAT) in plasma circulating cell-free DNA by nanoplate-based digital PCR in a pilot cohort including 29 BC-negative samples, 10 patients with non-muscle-invasive BC (NMIBC), and 7 patients with muscle-invasive BC (MIBC). Plasma ec-hASAT copy number was higher in patients with BC than in the BC-negative group (Mann–Whitney U test, *p* = 6.61 × 10^−7^). BC-negative samples ranged from 225 to 11,864 copies/µL plasma, whereas BC samples ranged from 1138 to 16,097 copies/µL plasma. ROC analysis yielded an AUC of 0.944 for discriminating BC from BC-negative samples. At an exploratory threshold of 2000 copies/µL plasma, sensitivity was 94.1% (16/17; exact 95% CI, 71.3–99.9%) and specificity was 89.7% (26/29; exact 95% CI, 72.6–97.8%). These preliminary data support plasma ec-hASAT as a candidate minimally invasive biomarker for BC detection, including NMIBC, and justify validation in larger prospective cohorts.

## 1. Introduction

Bladder cancer (BC) is one of the most common malignancies of the urinary tract and is characterized by a high recurrence rate; consequently, patients require long-term surveillance and repeated invasive procedures. Current global estimates indicate that bladder cancer ranks among the most common cancers worldwide, with more than 600,000 new cases and more than 220,000 deaths reported in 2022 [1].

BC is commonly stratified into non-muscle-invasive bladder cancer (NMIBC) and muscle-invasive bladder cancer (MIBC), which differ in biological behavior, prognosis, and therapeutic management [2]. NMIBC accounts for most newly diagnosed cases and often requires repeated follow-up, whereas MIBC is associated with more aggressive treatment strategies and poorer prognosis. Sensitive and accurate detection is therefore central to clinical management.

Cystoscopy and urinary cytology remain important methods for BC diagnosis and surveillance, even though both have substantial limitations. Cystoscopy is invasive and heavy for patients, whereas cytology has limited sensitivity, particularly for low-grade lesions [2,3]. Several liquid biopsy approaches have been investigated for BC, including circulating tumor cells, circulating cell-free DNA (cfDNA), urinary cell-free DNA, RNA, and protein biomarkers; however, no molecular test has yet replaced standard diagnostic procedures in routine clinical practice [4,5,6,7].

cfDNA is released into biological fluids through multiple processes, including cell death and active release, and may be associated with protein complexes or extracellular vesicles [8,9]. Repetitive DNA elements represent a large and biologically relevant fraction of circulating nucleic acids. Alu sequences, LINE-1 elements, and satellite DNA have all been proposed as informative components of liquid biopsy assays [10,11,12].

Human alpha-satellite DNA is a family of tandemly repeated DNA composed of approximately 171 bp monomeric repeat units, organized primarily in centromeric and pericentromeric regions and involved in centromere function and chromosomal stability [13,14]. Alterations in the expression or copy-number dynamics of satellite sequences have been described in several cancers, and nucleic acids derived from satellite sequences may reflect tumor-associated genomic instability and chromatin deregulation [15,16,17,18]. Based on these observations, we hypothesized that circulating extracellular alpha-satellite DNA may be enriched in the plasma of patients with BC and may represent a candidate minimally invasive biomarker.

Here, we report preliminary evidence obtained by digital PCR indicating increased plasma ec-hASAT copy number in patients with BC compared with BC-negative samples. The present manuscript examines the preliminary cohort included in this study and presents the diagnostic performance of ec-hASAT in discriminating patients with BC from BC-negative samples, while emphasizing the exploratory nature of the findings and the need for independent validation.

## 2. Results

### 2.1. Cohort Characteristics

The analyzed cohort comprised 29 BC-negative samples, 10 patients with NMIBC, and 7 patients with MIBC (Table 1). Samples without a BC diagnosis were assigned to the BC-negative group. This group included samples without a diagnosis of bladder cancer. It comprised non-oncological controls and samples annotated with non-BC malignancies. Therefore, BC-negative samples should not be interpreted as a homogeneous healthy-control group. Healthy/non-oncological controls were included as a defined subgroup within the BC-negative group. The specific composition of the BC-negative group is reported in Table 1.

The BC-negative group had a mean age of 60.1 years (range, 21–88), whereas patients with NMIBC and MIBC had mean ages of 76.7 years (range, 63–90) and 74.9 years (range, 53–87), respectively. The patient cohort was predominantly male, consistent with the epidemiology of BC; however, this pilot dataset was not intended to evaluate sex-specific diagnostic performance.

### 2.2. PCR Amplification Confirms the Presence of Plasma ec-hASAT Amplicons

**Figure 1:**
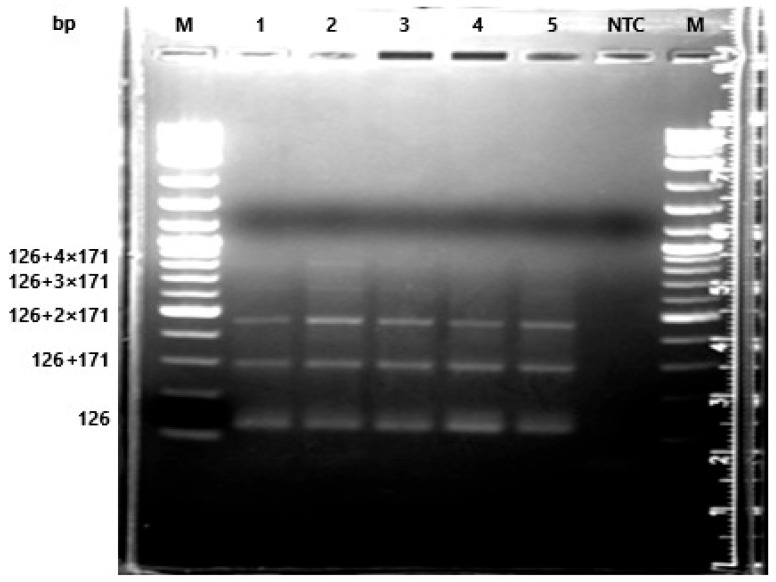
Representative agarose gel electrophoresis of hASAT PCR amplicons obtained from five purified plasma circulating cell-free DNA samples (lanes 1, 2: MIBC; lanes 3, 4: NMIBC; lane 5: BC-negative; NTC: no-template control). Molecular weight markers (lanes M) are shown on both sides of the gel (1 kb Plus DNA Ladder, New England Biolabs, Ipswich, MA, USA). The primers amplify a 126 bp hASAT fragment, and the higher-molecular-weight products are compatible with tandem alpha-satellite multimers separated by the 171 bp repeat unit. Amplicon molecular sizes (bp) are also displayed.

Standard PCR amplification using ec-hASAT-specific primers was performed as an orthogonal qualitative control before quantitative digital PCR analysis. A representative agarose gel showed amplification from five purified plasma circulating cell-free DNA samples, with the expected 126 bp product and additional bands compatible with the tandem organization of alpha-satellite repeats. The ladder-like profile is consistent with amplification products spaced by the 171 bp monomeric repeat length, supporting the presence of extracellular hASAT fragments in plasma up to pentameric elements (Figure 1).

### 2.3. Plasma ec-hASAT Copy Number Is Increased in Patients with Bladder Cancer

Nanoplate-based digital PCR showed a marked increase in plasma ec-hASAT copy number in patients with BC compared with the BC-negative group. In the study cohort, BC-negative samples ranged from 225 to 11,864 copies/µL plasma (three BC-negative samples exceeded 2000 copies/µL and were annotated as prostate- or ovarian cancer-related samples), whereas BC samples ranged from 1138 to 16,097 copies/µL plasma. The median ec-hASAT copy number was 846.5 copies/µL in the BC-negative group, 3060.5 copies/µL in NMIBC, and 3293.0 copies/µL in MIBC (Table 2).

**Figure 2:**
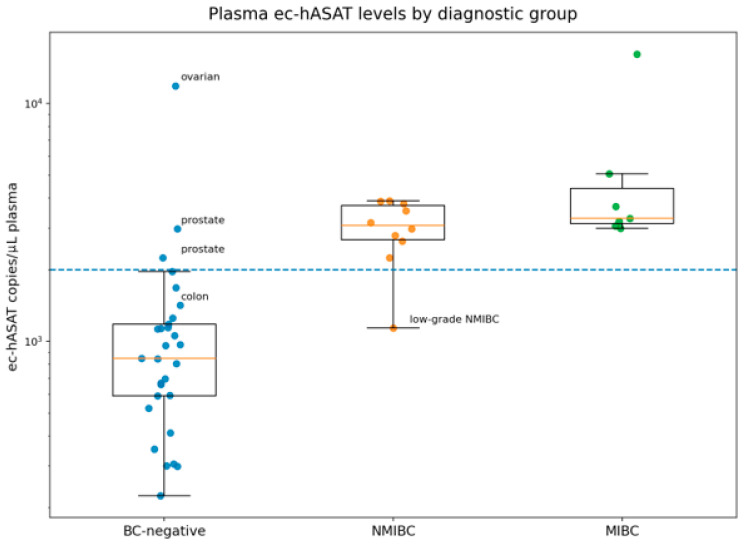
Plasma ec-hASAT levels in BC-negative samples and in patients with bladder cancer stratified by stage. Individual points represent samples. The dashed line indicates the exploratory threshold of 2000 copies/µL plasma. The y-axis is shown on a logarithmic scale to visualize both the control and tumor distributions. Kruskal–Wallis *p* = 3.14 × 10^−6^; all BC versus BC-negative *p* = 6.61 × 10^−7^; NMIBC versus MIBC *p* = 0.193.

The Kruskal–Wallis test showed a significant overall difference among the BC-negative, NMIBC, and MIBC groups (H = 25.34, *p* = 3.14 × 10^−6^). Pairwise analyses showed significant differences between the BC-negative group and patients with NMIBC (*p* = 7.61 × 10^−5^) and between the BC-negative group and patients with MIBC (*p* = 7.19 × 10^−6^). By contrast, ec-hASAT levels did not differ significantly between patients with NMIBC and MIBC (*p* = 0.193), indicating that the marker was increased in both disease stages in this preliminary cohort (Figure 2).

### 2.4. Exploratory Diagnostic Performance

**Figure 3:**
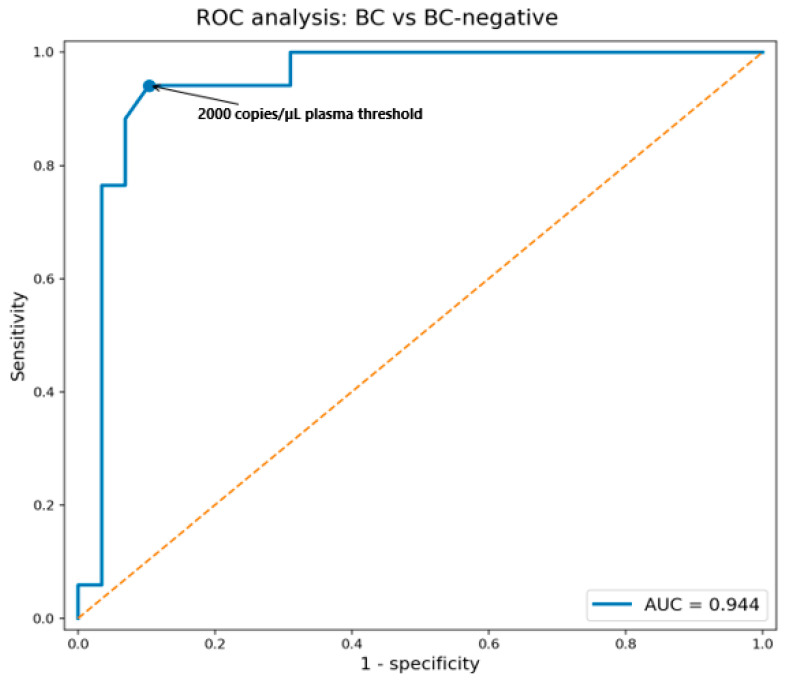
ROC analysis of plasma ec-hASAT copy for discrimination of patients with bladder cancer from BC-negative samples in the preliminary cohort. AUC = 0.944 for *n* = 17 patients with BC and *n* = 29 BC-negative samples. The highlighted point corresponds to the exploratory threshold of 2000 copies/µL plasma. The dashed line represents a baseline model with zero predictive ability. It shows the performance of a classifier that makes decisions by pure chance, i.e., at any point on this line, the probability of correctly identifying a positive case is exactly equal to the probability of incorrectly classifying a negative case, representing the absolute minimum standard for a useful predictive model.

ROC analysis yielded an AUC of 0.944 for discriminating patients with BC from BC-negative samples (Figure 3). At the preliminary threshold of 2000 ec-hASAT copies/µL plasma, sensitivity was 94.1% (16/17; exact 95% CI, 71.3–99.9%) and specificity was 89.7% (26/29; exact 95% CI, 72.6–97.8%).

Three BC-negative samples exceeded the 2000-copy/µL threshold and were annotated as prostate or ovarian carcinoma. A fourth BC-negative sample, annotated as colon carcinoma, did not exceed the threshold but showed a relatively high value that was close to it. These samples were retained in the BC-negative group because they were clinically negative for bladder cancer, which was the diagnostic endpoint of the study. Therefore, while these samples should primarily be considered clinical false-positive BC controls, they may also support the possibility that increased ec-hASAT is associated with other oncological conditions or broader states of genomic instability.

Conversely, one low-grade NMIBC sample was below the 2000-copy/µL threshold. Thus, the 2000-copy/µL cutoff remains exploratory and requires prospective validation including benign urological diseases, hematuria controls, inflammatory conditions and non-BC malignancies to determine whether ec-hASAT is a bladder-cancer-specific marker or a more general malignancy-associated marker.

## 3. Discussion

This pilot study provides preliminary evidence that plasma ec-hASAT copy number is increased in patients with bladder cancer compared with a BC-negative group. The increase was evident in both NMIBC and MIBC, suggesting that the signal is not limited to advanced muscle-invasive disease. These findings support the hypothesis that extracellular alpha-satellite DNA may reflect tumor-associated genomic instability or altered handling of repetitive DNA during bladder carcinogenesis.

The diagnostic analysis did not show complete separation between patients with BC and the BC-negative group. This is scientifically important because it avoids overestimating BC specificity and makes explicit that ec-hASAT may also be elevated in other oncological conditions. Future validation must include benign urological diseases, hematuria controls, inflammatory conditions, and non-BC malignancies to determine whether ec-hASAT is a bladder-cancer-specific marker or a more general malignancy-associated marker.

Further, therapeutic interventions may influence circulating ec-hASAT levels. Surgery, intravesical therapy, chemotherapy, radiotherapy, inflammation, tissue injury, and treatment-induced cell death could, in theory, increase the release of extracellular DNA, including repetitive DNA. The samples used in this study come from patients that were invited to enter into a more specialized checkup and plasma was collected before any clinical treatment and knowledge of the specific diagnosis. Future prospective studies should record treatment status, hematuria, inflammatory conditions and the timing of blood sampling relative to diagnostic or therapeutic procedures.

The elevated ec-hASAT values observed in BC-negative samples annotated as prostate, ovarian, or colon carcinoma are particularly important. These samples may represent biological positives for cancer-associated extracellular alpha-satellite DNA rather than analytical false positives. This interpretation is compatible with previous evidence that blood alpha-satellite RNA is increased in patients with metastatic castration-resistant prostate cancer [19]. Accordingly, the marker should currently be described as a candidate biomarker for BC identification with possible broader oncological relevance, rather than as a validated BC-specific diagnostic test.

The observation that patients with NMIBC showed elevated ec-hASAT levels remains clinically relevant because non-invasive approaches for early diagnosis and follow-up represent an important clinical need in BC. Although cystoscopy remains indispensable for diagnosis and surveillance, a blood-based marker could become useful if it could reliably identify patients requiring further urological evaluation, complement urinary tests, including urinary DNA methylation assays [20], or support post-treatment monitoring.

The biological basis of ec-hASAT release remains to be clarified. Alpha-satellite DNA is normally organized in large centromeric arrays and contributes to chromosomal segregation and chromatin architecture [13,14]. Cancer-associated genomic instability, aberrant expression of satellite repeats, altered chromatin organization, and DNA repair processes may influence the abundance of satellite-derived DNA in the extracellular compartment [15,16,17,18]. In parallel, apoptosis, necrosis, micronucleus formation, and the release of chromatin fragments may contribute to the extracellular appearance of alpha-satellite-derived DNA. Therefore, increased ec-hASAT in plasma may reflect a combination of tumor-associated chromosomal instability, altered satellite DNA turnover and the increased extracellular release of repetitive DNA.

From a diagnostic perspective, the AUC of 0.944 and the 2000-copy/µL threshold are encouraging but should not be overinterpreted. The cohort is small, retrospective, and imbalanced for age and sex. Future studies should predefine sampling, normalization units, exclusion criteria, and thresholds, and should include benign urological conditions, inflammatory conditions, hematuria controls, non-BC tumors, and longitudinal post-treatment samples.

An additional limitation is that the current dataset does not establish whether ec-hASAT levels correlate with tumor burden, recurrence risk, treatment response or longitudinal disease status. Prospective sampling before and after transurethral resection, intravesical therapy, cystectomy, or systemic therapy could clarify whether ec-hASAT is useful for monitoring therapeutic response or early recurrence.

Overall, the present data justify a larger validation study of plasma ec-hASAT as a candidate minimally invasive biomarker for BC identification and its exploration in other tumor types. This manuscript deliberately uses cautious terminology, because the observed performance must be reproduced in independent cohorts before clinical claims can be made.

## 4. Materials and Methods

### 4.1. Study Cohort and Sample Collection

Blood was collected in VACUETTE^®^ EDTA Tubes with gel separator (Greiner Bio-One, Kremsmünster, Austria) and processed within 1 h after venipuncture. Plasma was separated by two sequential centrifugation steps. The first centrifugation was performed at 5000 rpm for 5 min. Only a small aliquot of the plasma supernatant, corresponding to 200 microliters, was then transferred to a new tube and centrifuged again under the same conditions. Because alpha-satellite DNA is highly abundant in cellular genomic DNA, contamination from leukocytes or cellular debris is a relevant pre-analytical concern. The specific tubes used, the double-centrifugation protocol adopted and the timing for DNA extraction performed immediately after plasma separation avoiding freeze–thaw cycles were used in order to eliminate cellular genomic DNA in this retrospective pilot study.

Clinical information included BC status, disease stage (NMIBC or MIBC), tumor grade, sex, and age.

For the present exploratory analysis, the cohort was defined according to the clinical annotations and digital PCR values available for the study samples. Samples without a BC diagnosis were analyzed as BC-negative.

### 4.2. Isolation of Circulating Cell-Free DNA

Circulating cell-free DNA was isolated from plasma after 30 min proteinase K digestion at 55 °C and purified using the QIAamp MinElute ccfDNA Kits: Plasma & Serum cfDNA (QIAGEN, Hilden, Germany), according to the manufacturer’s instructions. The proteinase K digestion step was retained because direct plasma dilution did not yield reliable PCR amplification in preliminary experiments, suggesting that at least part of the extracellular DNA may be associated with protein-containing complexes, although this issue requires further dedicated mechanistic experiments. For all samples, cell free DNA was purified from 100 µL of plasma and eluted in 80 µL of nuclease-free water. The purified DNA was diluted 1:10 and 10 µL of diluted DNA was used in a 15 µL final digital PCR reaction. Twelve microliters were loaded into the nanoplate; this loaded volume corresponded to 1 µL of plasma-equivalent input DNA. The QIAcuity Software Suite and Control Software version 3.2 automatically reported ec-hASAT-positive target units per microliter of digital PCR reaction. Because all samples were processed using the same plasma input, elution volume, dilution factor, reaction volume, and loaded volume, the values were directly comparable across the cohort. The present pilot study focused on absolute digital PCR quantification of the ec-hASAT target and did not systematically include fluorometric quantification of total cfDNA or fragment-size distribution analysis. Therefore, total cfDNA yield and cfDNA fragment integrity could not be used as quality-control parameters in this retrospective dataset.

### 4.3. Quantification of ec-hASAT by Digital PCR

Digital PCR was selected instead of qPCR because it enables absolute quantification without external standard curves, provides partition-based counting of target-positive reactions using Poisson statistics, and is suitable for low-input circulating DNA.

Because alpha-satellite DNA are organized as tandem repeats, the digital PCR assay should be interpreted as measuring amplifiable ec-hASAT target-positive DNA units rather than necessarily measuring individual alpha-satellite monomers. If multiple ec-hASAT targets are physically linked on the same plasma DNA molecule, they may enter the same digital PCR partition and be counted as a single positive monomer and, vice versa, multiple monomers can be counted as a single positive molecule. Controlled restriction digestion or fragmentation was not applied in this study because fundamentally alpha-satellite repeats are heterogeneous, and identifying a restriction enzyme able to reproducibly fragment all relevant repeat arrays without introducing bias is almost impossible. Furthermore, incomplete digestion could introduce an additional source of variability. The aim of the present study was to quantify the native amplifiable ec-hASAT-positive fraction recovered from plasma under standardized conditions.

The absolute quantification of ec-hASAT was performed using a nanoplate-based digital PCR platform (QIAcuity 2-plex; QIAGEN, Hilden, Germany). Reactions contained QIAcuity 3× EvaGreen PCR Master Mix (QIAGEN, Hilden, Germany), primer mix, RNase-free water, and 1 µL of template DNA. Twelve microliters of the reaction mixture were transferred to a 24-well 8.5 K nanoplate and sealed with the corresponding rubber seal. Digital PCR reactions were prepared in a final volume of 15 µL. For all samples, purified plasma DNA was diluted 1:10, and 10 µL of diluted DNA was added to each reaction. Because DNA was purified from 100 µL of plasma and eluted in 80 µL, the 12 µL loaded into the nanoplate corresponded to 1 µL of plasma-equivalent input DNA. Total cfDNA concentration was not systematically measured since these targets are usually below the detection thresholds for fluorometric/spectrophotometric quantification; therefore, template input could not be consistently reported as ng/µL for the full cohort. The ec-hASAT primers were used at a final concentration of 0.2 µM.

The ec-hASAT primer sequences were forward 5′-CACTCTTTTTGTAGAATCTGC-3′ and reverse 5′-AATGCACACATCACAAAGAAG-3′. The ec-hASAT primers were designed to amplify a 126 bp region within the human alpha-satellite repeat monomers. These primers specifically target the conserved parts of the sequence intended to amplify the majority of the satellites originating from (peri)centromeric regions, as demonstrated here (Figure 1) and as previously described by Ljubić et al., [19].

The amplification protocol consisted of enzyme activation at 95 °C for 2 min, followed by 40 cycles of 95 °C for 15 s, 60 °C for 15 s, and 72 °C for 15 s, with a final step at 4 °C. For samples with two performed digital PCR measurements, the median of the two values was used for analysis.

Positive partitions were quantified by the QIAcuity software using Poisson statistics. The software output was reported as ec-hASAT copy concentration, expressed as copies/µL of digital PCR reaction. Under the standardized workflow described above, the 12 µL reaction volume loaded into the nanoplate corresponded to 1 µL of plasma-equivalent input DNA. Therefore, all samples were compared under identical plasma-equivalent input conditions. No-template controls were included to monitor possible contamination.

Digital PCR runs were accepted only when nanoplate wells passed the instrument quality-control criteria and no-template controls showed no amplification or only background signal below the positivity threshold. Fluorescence thresholds were set using the QIAcuity software and manually inspected to verify separation between positive and negative partitions. Replicate measurements were reviewed for concordance.

No additional manual positive-partition cutoff was applied beyond the QIAcuity software quality-control and Poisson-based quantification workflow.

### 4.4. PCR Amplification and Amplicon Detection

Endpoint PCR was performed in 25 µL reactions containing 12.5 µL Taq Green Master Mix, ec-hASAT primers F (5′-CACTCTTTTTGTAGAATCTGC-3′) and R (5′-AATGCACACATCACAAAGAAG-3′) at a final concentration of 0.2 µM, 1 µL purified plasma DNA template, and nuclease-free water. Because total DNA concentration was not systematically measured for all samples, endpoint PCR template input is reported as volume rather than ng.

PCR was performed using a Bio-Rad T100 thermal cycler (Bio-Rad Laboratories, Hercules, CA, USA). The thermal cycling program consisted of 5 min at 95 °C for initial denaturation, followed by 30 cycles of 30 s at 95 °C for denaturation, 30 s at 50 °C for annealing, and 60 s at 72 °C for extension, and 5 min at 72 °C for final extension. PCR products were examined by electrophoresis at 100 V for 60 min in a 1% (*w*/*v*) agarose gel in 1× TAE buffer. A 1 kb DNA ladder was used as the marker. The electrophoretic gel was immersed in ethidium bromide for 30 min and examined under UV light.

### 4.5. Statistical Analysis

Continuous variables are summarized using medians and interquartile ranges unless otherwise specified. The Shapiro–Wilk test was used to test data normality. Differences among the BC-negative, NMIBC, and MIBC groups were assessed using the Kruskal–Wallis test. Pairwise comparisons were performed using two-sided Mann–Whitney U tests. Diagnostic performance for BC versus BC-negative samples was assessed by ROC analysis and summarized using the AUC. Sensitivity and specificity were calculated at the exploratory threshold of 2000 copies/µL plasma, with exact binomial 95% confidence intervals. This cut-off was estimated as optimal during ROC curve plotting by generating probabilities and calculating rates. It designates the threshold for the point on the curve that is geographically closest to the perfect classification point. In the context of diagnostics, it is also driven by the cost of errors (e.g., maximizing sensitivity to avoid missing positive cases). Additionally, the 2000 copies/µL plasma-equivalent input threshold was selected as an exploratory working threshold based on the distribution observed in the pilot cohort. It was not predefined in an independent validation cohort and should not be interpreted as a clinically validated diagnostic cut-off. Because cut-off selection and performance estimation were performed on the same dataset, sensitivity and specificity may be optimistic and are reported only as exploratory apparent performance. Analyses were performed in Python (version 3.10) using SciPy (version 1.7.3) and scikit-learn (version 1.0.2).

## 5. Conclusions

Plasma extracellular alpha-satellite DNA was markedly increased in patients with bladder cancer compared with BC-negative samples in this preliminary cohort analyzed by digital PCR. The presence of elevated values in some BC-negative samples annotated as non-BCs provides biologically relevant evidence that ec-hASAT may also be elevated in other oncological conditions. These findings support further validation of ec-hASAT as a candidate minimally invasive biomarker for BC identification and, more broadly, as a possible marker of cancer-associated genomic instability. Larger prospective studies are required before clinical implementation and before definitive diagnostic claims can be made.

## 6. Patents

I.F. is listed as inventor and holder of Italian patent application No. 102023000007245, filed on 14 April 2023, entitled “Novel circulating biomarker in human plasma (hASAT), capable of discriminating patients affected by cancer from healthy subjects, useful for the early diagnosis of bladder cancer and for monitoring response to therapeutic treatments”. The patent was granted on 29 April 2025.

## Figures and Tables

**Table 1:** Demographic and clinical characteristics of the preliminary cohort.

Group	Characteristic	Value
BC-negative group	*n*	29
	male/female	14/15
	mean age/years	60.1
	age min–max/years	21–88
	healthy/non-BC malignancy	25/4
NMIBC patients	*n*	10
	low (LG)/high grade (HG)	4/6
	male/female	9/1
	mean age/years	76.7
	age min–max/years	63–90
MIBC patients	*n*	7
	low (LG)/high grade (HG)	0/7
	male/female	7/0
	mean age/years	74.9
	age min–max/years	53–87

NMIBC, non-muscle-invasive bladder cancer; MIBC, muscle-invasive bladder cancer; LG, low grade; HG, high grade. The BC-negative group includes samples without a BC diagnosis, including samples annotated as other cancers or non-malignant conditions.

**Table 2:** Distribution of plasma ec-hASAT copy number in the preliminary cohort. IQR, interquartile range. Values are reported as ec-hASAT copy numbers per microliter of plasma derived from the digital PCR output used for the preliminary analysis.

Group	*n*	Median Copies/µL	IQR	Range
BC-negative	29	846.5	588–1182	225–11,864
NMIBC	10	3060.5	2669–3718.4	1138–3895
MIBC	7	3293	3128–4379.5	2980–16,097
All BC	17	3200	2974–3780	1138–16,097

## Data Availability

The anonymized dataset and preprocessing notes supporting the findings of this study are available from the corresponding author upon reasonable request, subject to applicable ethical and privacy restrictions.

## References

[1] Bray F., Laversanne M., Sung H., Ferlay J., Siegel R.L., Soerjomataram I., Jemal A. (2024). Global cancer statistics 2022: GLOBOCAN estimates of incidence and mortality worldwide for 36 cancers in 185 countries. CA Cancer J. Clin..

[2] Sanli O., Dobruch J., Knowles M.A., Burger M., Alemozaffar M., Nielsen M.E., Lotan Y. (2017). Bladder cancer. Nat. Rev. Dis. Prim..

[3] Zhu C.Z., Ting H.N., Ng K.H., Ong T.A. (2019). A review on the accuracy of bladder cancer detection methods. J. Cancer.

[4] Lodewijk I., Duenas M., Rubio C., Munera-Maravilla E., Segovia C., Bernardini A., Teijeira A., Paramio J.M., Suárez-Cabrera C. (2018). Liquid biopsy biomarkers in bladder cancer: A current need for patient diagnosis and monitoring. Int. J. Mol. Sci..

[5] Herranz R., Oto J., Plana E., Fernandez-Pardo A., Cana F., Martinez-Sarmiento M., Vera-Donoso C.D., Espana F., Medina P. (2021). Circulating cell-free DNA in liquid biopsies as potential biomarker for bladder cancer: A systematic review. Cancers.

[6] Riethdorf S., Soave A., Rink M. (2017). The current status and clinical value of circulating tumor cells and circulating cell-free tumor DNA in bladder cancer. Transl. Androl. Urol..

[7] Tse R.T., Zhao H., Wong C.Y., Cheng C.K., Kong A.W., Peng Q., Chiu P.K., Ng C.F., Teoh J.Y. (2021). Urinary cell-free DNA in bladder cancer detection. Diagnostics.

[8] Corcoran R.B., Chabner B.A. (2018). Application of cell-free DNA analysis to cancer treatment. N. Engl. J. Med..

[9] Tutanov O., Shtam T., Grigorieva A., Tupikin A., Tsentalovich Y., Tamkovich S. (2022). Blood plasma exosomes contain circulating DNA in their crown. Diagnostics.

[10] Gezer U., Bronkhorst A.J., Holdenrieder S. (2022). The utility of repetitive cell-free DNA in cancer liquid biopsies. Diagnostics.

[11] Grabuschnig S., Soh J., Heidinger P., Bachler T., Hirschboeck E., Rodriguez I.R., Schwendenwein D., Sensen C.W. (2020). Circulating cell-free DNA is predominantly composed of retrotransposable elements and non-telomeric satellite DNA. J. Biotechnol..

[12] Ozgur E., Mayer Z., Keskin M., Yoruker E.E., Holdenrieder S., Gezer U. (2021). Satellite 2 repeat DNA in blood plasma as a candidate biomarker for the detection of cancer. Clin. Chim. Acta.

[13] Ugarkovic D., Sermek A., Ljubic S., Feliciello I. (2022). Satellite DNAs in health and disease. Genes.

[14] McNulty S.M., Sullivan B.A. (2018). Alpha satellite DNA biology: Finding function in the recesses of the genome. Chromosome Res..

[15] Ting D.T., Lipson D., Paul S., Brannigan B.W., Akhavanfard S., Coffman E.J., Contino G., Deshpande V., Iafrate A.J., Letovsky S. (2011). Aberrant overexpression of satellite repeats in pancreatic and other epithelial cancers. Science.

[16] Bersani F., Lee E., Kharchenko P.V., Xu A.W., Liu M., Xega K., MacKenzie O.C., Brannigan B.W., Wittner B.S., Jung H. (2015). Pericentromeric satellite repeat expansions through RNA-derived DNA intermediates in cancer. Proc. Natl. Acad. Sci. USA.

[17] Kishikawa T., Otsuka M., Yoshikawa T., Ohno M., Yamamoto K., Yamamoto N., Kotani A., Koike K. (2016). Quantitation of circulating satellite RNAs in pancreatic cancer patients. JCI Insight.

[18] Feliciello I., Picariello O., Chinali G. (2006). Intra-specific variability and unusual organization of the repetitive units in a satellite DNA from Rana dalmatina: Molecular evidence of a new mechanism of DNA repair acting on satellite DNA. Gene.

[19] Ljubic S., Sermek A., Prgomet Secan A., Prpic M., Jaksic B., Murgic J., Frobe A., Ugarkovic D., Feliciello I. (2022). Alpha satellite RNA levels are upregulated in the blood of patients with metastatic castration-resistant prostate cancer. Genes.

[20] Hentschel A.E., Beijert I.J., Bosschieter J., Kauer P.C., Vis A.N., Lissenberg-Witte B.I., van Moorselaar R.J.A., Steenbergen R.D.M., Nieuwenhuijzen J.A. (2022). Bladder cancer detection in urine using DNA methylation markers: A technical and prospective preclinical validation. Clin. Epigenet..

